# Prokaryotic taxa play keystone roles in the soil microbiome associated with woody perennial plants in the genus *Buxus*

**DOI:** 10.1002/ece3.5614

**Published:** 2019-08-26

**Authors:** Nicholas LeBlanc, Jo Anne Crouch

**Affiliations:** ^1^ Mycology and Nematology Genetic Diversity and Biology Laboratory U.S. Department of Agriculture, Agricultural Research Service Beltsville MD USA; ^2^ ARS Research Participation Program Oak Ridge Institute for Science and Education Oak Ridge TN USA

**Keywords:** archaea, bacteria, boxwood, fungi, soil microbiome

## Abstract

The microbiome associated with ornamental plants has largely been neglected, despite its potential for impacting plant health. This work characterized the composition, diversity, and microbial co‐associations in the soil microbiome associated with species and cultivars of plant in the genus *Buxus* (common name boxwood), a group of woody perennial shrubs commonly used in residential landscapes and found in native ecosystems. Soil was collected from 82 individual curated boxwood accessions at the U.S. National Arboretum National Boxwood Collection. Amplicon libraries targeting archaea, bacteria, and fungi were generated and sequenced using the Illumina MiSeq platform. Identification of individual sequence variants resulted in 275 archaeal, 15,580 bacterial, and 7,525 fungal taxa. Neither spatial distance among samples nor association with different types of boxwood were significant predictors of soil microbiome composition. However, archaeal and bacterial diversity was significantly different in soil from distinct types of boxwood. Co‐association networks indicated that archaea and bacteria show greater evidence of being keystone taxa than fungi. Overall, this work demonstrates the potential for targeting specific keystone taxa to shift the soil microbiome associated with these boxwood accessions and that planting different species or cultivars in the landscape may shift the diversity of prokaryotic microorganisms.

## Graphical abstract

Archaea, bacteria, and fungi in soil associated with perennial boxwood shrubs were characterized using meta‐barcoding. Archaeal and bacterial diversity was influenced by boxwood species/cultivar and prokaryotes showed evidence of being keystone taxa in the soil microbiome.

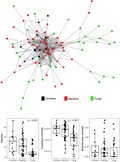

## 1 INTRODUCTION

Soilborne prokaryotic and eukaryotic microorganisms play essential functional roles that influence the growth of plants in natural and managed ecosystems. The importance of these microbial groups has motivated efforts to understand their ecology in global ecosystems and develop ways to manipulate their impacts on plants through management or plant breeding (Bakker, Manter, Sheflin, Weir, & Vivanco, 2012; Fierer, 2017; Walters et al., 2018). Despite the potential to leverage the microbial diversity in soil to influence plant growth, aside from work in turfgrass (Beirn et al., 2017; Crouch, Carter, Ismaiel, & Roberts, 2017), relatively little research has focused on plants that are cultivated for their ornamental traits.

Plants in the genus *Buxus* (commonly called boxwood) have a long history of cultivation as woody ornamental shrubs (Batdorf, 2004). As a group, these plants are a substantial economic sector of for the horticultural industry, most recently valued at 120 million dollars per year in the United States (USDA‐National Agricultural Statistics Service Census of Agriculture 2014 reports). Three species of boxwood are commercially important for the horticulture industry: *B. sempervirens* (European boxwood, the most common), *B. microphylla,* and *B. sinica* (Japanese and Korean boxwood, respectively, Niemiera, 2012). However, approximately 90 species of boxwood have been described. This broader genetic diversity is an essential resource for the genetic improvement of these plants (Pooler, 2017) and an important component of global terrestrial ecosystems (Domenico, Lucchese, & Magri, 2012).

Though boxwood are notable for their resistance toward plant diseases (Batdorf, 2004), recent emergence of the disease boxwood blight poses a significant threat to the health of these plants (LeBlanc, Salgado‐Salazar, & Crouch, 2018). Currently, there are few management options for effectively controlling boxwood blight (Palmer & Shishkoff, 2014) and the most widely grown boxwood are highly susceptible to the disease (LaMondia & Shishkoff, 2017; Shishkoff, Daughtrey, Aker, & Olsen, 2015). In addition, the more recent emergence of the box tree moth insect pest has further complicated the ability to maintain healthy boxwood in native ecosystems and urban landscapes (Kenis, Nacambo, Leuthardt, Di Domenico, & Haye, 2013). Considering the limited options for controlling boxwood blight and the box tree moth, leveraging the soil microbiome to suppress the causal pathogen of boxwood blight in soil or induce system resistance in plant foliage may serve as alternative solutions for disease and insect control.

The purpose of this work was to perform a descriptive study of variation in the prokaryotic and eukaryotic components of the soil microbiome associated with one of the most diverse collections of boxwood germplasm in the United States. This germplasm collection is maintained at the U.S. National Arboretum and serves as a resource for boxwood improvement (Pooler, 2017), including the identification of genetic resistance to plant diseases (LaMondia & Shishkoff, 2017; Shishkoff et al., 2015) and insect pests (d'Eustachio & Raupp, 2001). Focusing on this collection, we addressed three basic questions regarding the ecology of the soil microbiome associated with these plants: (a) does variation in plant genetic background influence the soil microbiome, (b) is there evidence of microbial dispersal limitation, and (c) do certain taxonomic groups show greater evidence of being keystone taxa in the soil microbiomes associated with this collection of plant germplasm? By focusing on these questions, this work provides the first step toward developing understanding the soil microbiome associated with boxwood and a platform for future research focused on developing applications for improving the health of these plants.

## 2 MATERIALS AND METHODS

### 2.1 Field sampling

**Figure 1:**
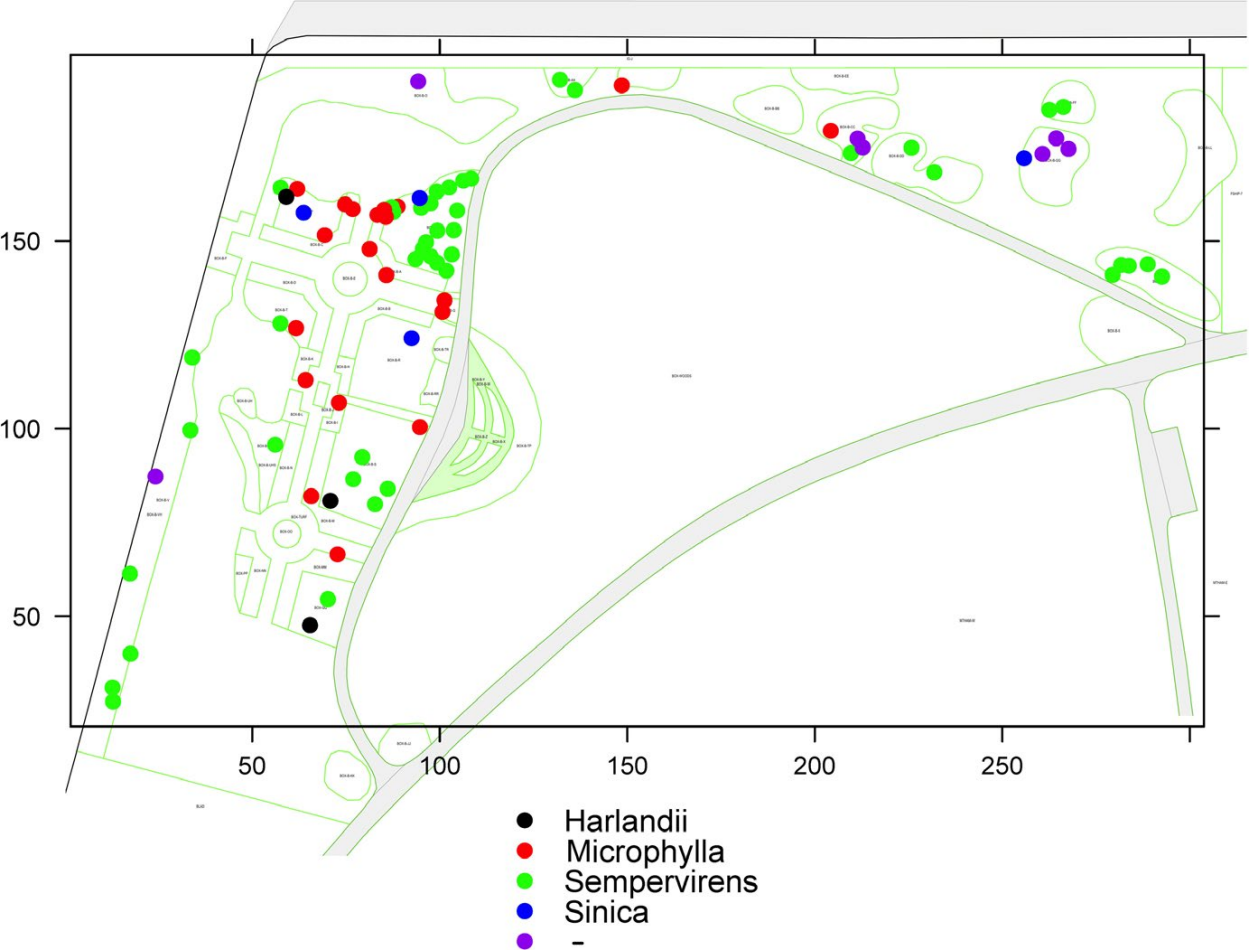
Map of soil samples collected at different landscape beds at the U.S. National Arboretum (USNA) National Boxwood Collection. Points represent spatial location of the samples within the collection. Color codes correspond to the species of boxwood from which the sample was collected and “‐” indicates unresolved subgenus classification. Units are in meters and the data are overlaid on a map of the USNA National Boxwood Collection. Different green segments in the map are approximate borders of different garden beds and the thicker gray lines represent service roads

Field sampling was performed at the U.S. National Arboretum (USNA) National Boxwood Collection in Washington, DC, USA (https://www.usna.usda.gov/discover/gardens-collections/boxwood-collection) in June 2016. Plants at this site are cared for by a full‐time professional horticulturist, and garden beds are mulched seasonally using mulch generated on‐site at the USNA. Soil was sampled from the upper 20 cm of soil of 82 individual boxwood plants. Soil was collected using a 15.9 mm diameter hand‐held auger positioned between 13 and 26 cm from the boxwood trunk, based on ability of the auger to penetrate the soil and accessibility under the canopy. Among the 82 sampled plants, 75 have been formally classified by the USNA, including *Buxus harlandii* (*n* = 3), *B. microphylla* (*n* = 20), *B. sempervirens* (*n* = 48), and *B. sinica* (*n* = 4; Table S1). Due to the abundance of *B. sempervirens* in the collection, 10 cultivars of this species were sampled with a minimum of three replicates. Where possible, replicates of individual boxwood species or *B. sempervirens* cultivars were selected from different beds within the National Boxwood Collection (see Figure 1 for spatial arrangement of soil samples within the USNA). Soil samples were processed to remove all but the section of the soil core occupied by boxwood roots to a depth of 7 cm. Soil was passed through a #20 screen (Newark Wire Cloth Co., Newark, NJ) to remove root debris and stored at −20°C until DNA extraction.

### 2.2 Amplicon library sequencing

DNA was extracted from the 82 soil samples using the MoBio PowerSoil^®^ kit (Qiagen, MD, USA). Amplicon libraries were generated from extracted DNA using primers targeting 16S rRNA for archaea and bacteria and the internal transcribed spacer (ITS) region for fungi (Table S2). Taxon‐specific primers were appended with Illumina‐compatible adapter sequences and variable bases at the 5′ ends to introduce sequence heterogeneity as described in Beirn et al. (2017). Each library included a negative control reaction using sterile water as a template.

Two rounds of PCR were used, the first to create taxon‐specific libraries from soil DNA and the second to tag the amplicons with Illumina Nextera indices and flow cell compatible sequences. All PCR reactions were performed using a C1000™ Thermal Cycler (Bio‐Rad, CA, USA). The first round of PCR used 25 μl reactions with the following ingredients: 5 μl NEB Taq 5X Master Mix (New England Biolabs, 0.5 μl forward and reverse primers (10 μM), 18.5 μl water, and 0.5 μl DNA (10 ng/μl). Reactions were run with the following program: 30 s 95°C, 34 cycles of 30 s 95°C, 55°C, 68°C, followed by a final extension for 5 min at 68°C. Amplification was quantified using a QIAxcel capillary electrophoresis system (Qiagen, CA, USA). Taxon‐specific libraries were pooled in equal molar concentration for each sample, cleaned using ZR‐96 DNA Clean and Concentrator™ purification columns (Zymo Research, CA, USA), and used as a template for the second round of PCR. The second round of PCR used 40 μl reactions with the following ingredients: 8 μl NEB Taq 5X Master Mix, 10 μl of each i5 and i7 Nextera indexes, 7 μl of water and 15 μl of template. Reactions were run with the following program: 3 min at 72°C, 30 s at 95°C, 12 cycles of 10 s at 95°C, 30 s at 55°C, 30 s at 68°C, followed by a final extension step for 5 min at 68°C. Amplicons were purified as described above, quantified using a Qubit fluorometer, and pooled in equal molar concentrations. Libraries were sequenced in a single run with a 30% Phi‐X spike‐in using 301 bp paired‐end sequencing v3 chemistry on a MiSeq sequencer (Illumina, CA, USA). Raw sequence data are deposited at NCBI's Sequence Read Archive as BioProject accession PRJNA503120.

### 2.3 Sequence variant identification and classification

Adapter traces and primer sequences were removed using cutadapt v1.14. For each sample, libraries targeting the three microbial groups were de‐multiplexed prior to sequence variant identification. Sequence variants were used instead of operational taxonomic units (OTUs) based on their advantages of reproducibility and biological interpretation (discussed by Callahan, McMurdie, and Holmes (2017) and Knight et al. (2018)). Sequence variants were identified using the Divisive Amplicon Denoising Algorithm (DADA2) as implemented in R v3.5.1 (Callahan et al., 2016; R Development Core Team, 2015). This algorithm models and corrects sequence errors from Illumina data generated from amplicon libraries and has increased accuracy relative to commonly used OTU‐based pipelines (Callahan et al., 2016). Prior to use of DADA2, fastq files were filtered to remove sequences with ambiguous nucleotides and filtered sequences were truncated to 240 bp. As paired‐end sequences did not overlap for the 16S rRNA archaeal and bacterial libraries, only forward reads were used in further steps. Sequence error rates were estimated and corrected to identify individual sequence variants using default parameters of the DADA2 algorithm (see online methods in Callahan et al., 2016). Chimeras were identified and removed using the *isBimera* function in the DADA2 package, which identifies chimeras as sequences that are derived from two different sequence variants.

Taxonomic classification of the sequence variants was performed using the Ribosomal Database Project (RDP) classifier (Wang, Garrity, Tiedje, & Cole, 2007) implemented in mothur v1.39.5 (Schloss et al., 2009). The SILVA database release number 128 was used for bacterial data, Greengenes 2013 database for archaeal data (poor taxonomic resolution for the predominant type of archaea in these data prevented the use of the SILVA database), and the UNITE version 6 database for fungal data (Schloss et al., 2009). Sequence variants classified outside of the targeted microbial groups or found in the negative controls were removed prior to further analysis. All subsequent analyses used sequence variants and abundance of sequences classified to a given variant.

### 2.4 Statistical analyses

Statistical analyses were performed in R v3.5.1 (R Development Core Team, 2015). All statistical analyses treated sequence variants as the taxonomic unit. Variation in sequence depth among samples was accounted for by rarefying data matrices to the sample with the fewest number of sequences. One‐way analysis of variance (ANOVA) was used to test for significant differences in taxonomic richness among the three microbial groups. Additional statistical analyses treated each microbial group independently, and principal coordinate analysis was performed using Bray–Curtis distances to evaluate evidence for compositional differences among soil samples from different boxwood species. A Mantel test was used to determine if there was a linear relationship between dissimilarity among microbial communities and physical distance (i.e., distance‐decay relationship) among sample locations. Community dissimilarity was calculated based on Bray–Curtis distance, and physical distance was calculated as Euclidean distances between the sampled plant locations at the USNA National Boxwood Collection (Figure 1). Both analyses were performed using R package vegan (Oksanen et al., 2018).

The remaining statistical tests only included data from the 75 soil samples from boxwood plants whose taxonomic identity was resolved below the genus level. Analysis of similarities (ANOSIM) was used to test for significant differences in the composition of the microbial groups among boxwood species and *B. sempervirens* cultivars using Bray–Curtis dissimilarity calculated from rarefied sequence abundance data. The R package DESeq2 was used to test for significant differences in the abundance of individual taxa among boxwood species and *B. sempervirens* cultivars (Love, Huber, & Anders, 2014). Taxa found in only one sample were not included and comparisons among boxwood species and cultivars were treated as independent analyses. Significant variation among treatments (boxwood type) was tested using likelihood ratio tests and significance values were adjusted for multiple comparisons using the Benjamini–Hochberg false discovery method. One‐way analysis of variance (ANOVA) was used to test for significant differences in Shannon diversity (*H*) among boxwood species and cultivars of *B. sempervirens*. Post hoc comparisons were made using Tukey's HSD test. Except where more stringent values are noted, *p*‐values in all analyses were considered significant when <.05.

Co‐association networks were generated using the SparCC python module (Friedman & Alm, 2012) and analyzed in the R environment. Networks were generated from the 82 samples based on correlations in sequence abundance among archaeal, bacterial, and fungal taxa. Pearson correlations were calculated among taxa found in more than 41 (50%) soil samples. Pseudo‐*p*‐values were calculated based on 100 replications. Correlations among taxa were considered significant when represented by coefficients >0.4 or <−0.4 and with pseudo‐*p*‐values <.01. Taxa that had pairwise connections but were not connected to any other taxa were not used to calculate summary statistics for the network. Three network summary statistics were calculated using the R package igraph (Gabor & Nepusz, 2006). Network *degrees* was calculated as the number of network edges leading to a node (i.e., taxon), *betweenness centrality* was calculated based on how often a node falls on the shortest path between nodes, and *closeness centrality* was calculated as the mean distance from a node to all other network nodes. Networks were visualized using the GGally (Schloerke et al., 2018) package in R. Differences in the network statistics among the three microbial groups were tested using one‐way analysis of variance (ANOVA), and post hoc comparisons were made using Tukey's HSD test.

## 3 RESULTS

### 3.1 Sequence output and taxonomic classification

**Figure 2:**
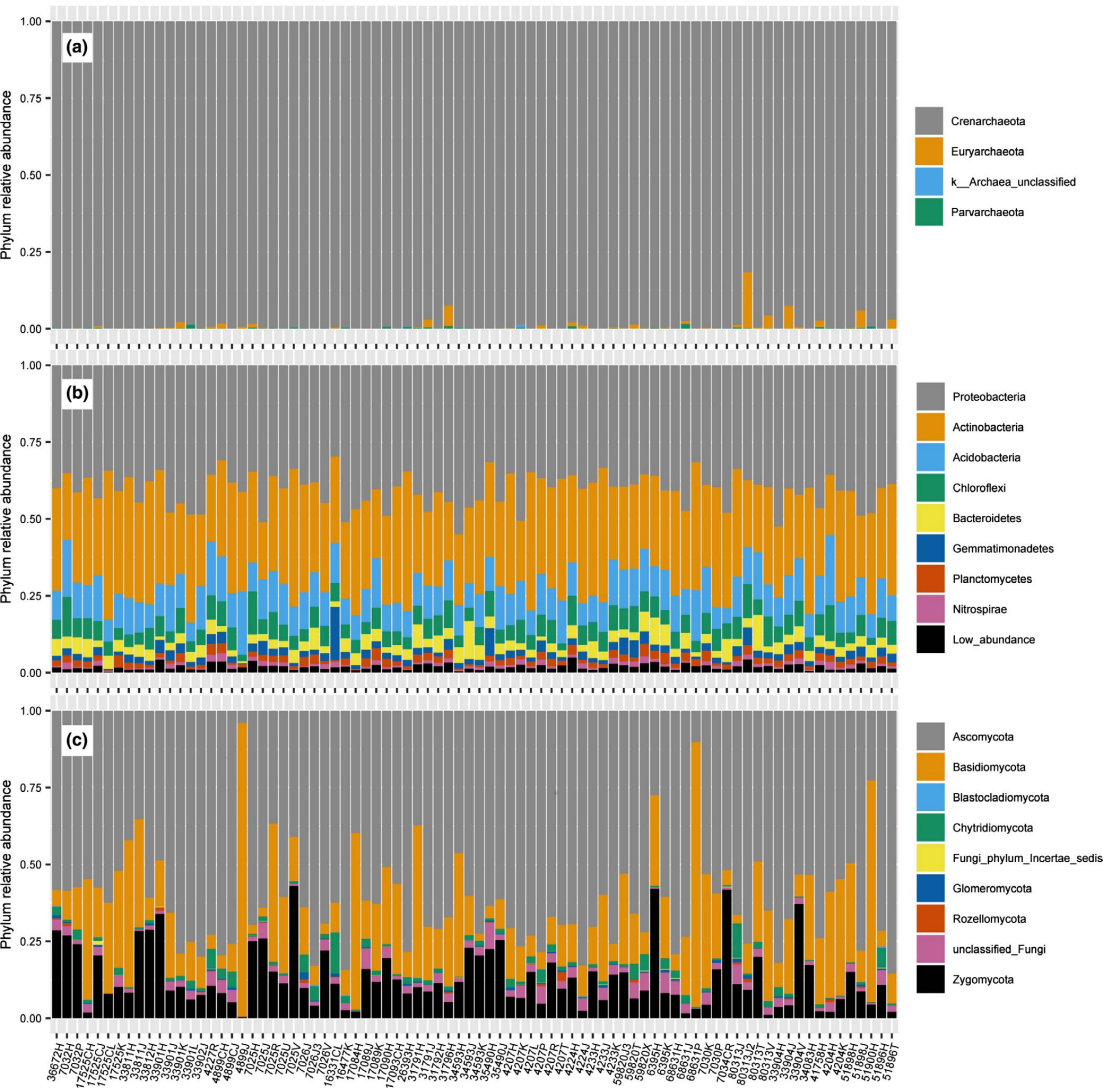
Relative abundance of microbial taxa in the soil microbiome. Data are summarized at the phylum level. Labels on the *x*‐axis represent the soil samples collected from the 82 boxwood accessions at the USNA National Boxwood Collection. The relative abundance for archaea (a), bacteria (b), and fungi (c) is on the *y*‐axis. Low abundance bacterial classification represents 36 phyla, each with less than 1% representation in the sequence data. Samples from the 82 boxwood germplasm accessions are on the *x*‐axis and relative abundances of different phyla are color‐coded. Low abundance bacterial classification represents 36 bacterial phyla, each with less than 1% representation in the sequence data

Following identification of sequence variants (hereafter referred as taxa) and removal of sequence data from nontarget organisms, a total of 8,370,868 sequences were classified within the three target groups, archaea, bacteria, or fungi. Among this total sequence output, there was variation in sequence depth among individual samples. The smallest sample was represented by 19,940 sequences and the largest by 386,074 sequences (Mean = 102,084; *SD* = 65,640). The number of taxa varied by an order of magnitude among the three microbial groups across the 82 samples. Archaea were represented by 275 taxa, bacteria by 15,580 taxa, and fungi by 7,525 taxa. One‐way ANOVA followed by post hoc comparisons indicated the number of taxa was significantly different among the three microbial groups (*F*_1,2_ = 193.1, *p* < .001; TukeyHSD, *p* < .001). At the phylum level, 99% of the archaea data were classified in the Crenarchaeota. In contrast, bacteria were classified in 44 different phyla and fungi in eight phyla (Figure 2).

### 3.2 Predictors of soil microbiome composition

The composition of archaea, bacteria, and fungi in soil was not significantly influenced by spatial distance among samples or the presence of different types of boxwood. Based on principal coordinate analysis, there was no clear differentiation within the three microbial groups among different boxwood species (data not shown). In addition, analysis of similarity (ANOSIM) tests showed no significant differences in composition within the three microbial groups in soil samples from different boxwood species or *B. sempervirens* cultivars (Table 1). Comparison of distance matrices from the meta‐barcoding data and a distance matrix constructed from *x*‐ and *y*‐coordinates of the mapped sampling locations (Figure 1) showed there was no significant relationship between microbiome composition and physical distance among sample locations. This was observed for all the samples or those collected only from the species *B. sempervirens* (Table 1).

**Table 1:** Tests for boxwood species and cultivar type and spatial distance as predictors of soil microbiome composition

Test	Archaea	Bacteria	Fungi
ANOSIM species (*p*‐value)^a^	0.092 (.056)	0.063 (.158)	0.061 (.176)
ANOSIM cultivar (*p*‐value)^b^	−0.028 (.618)	−0.022 (.598)	−0.05 (.741)
Mantel species (*p*‐value)^c^	−0.061 (.888)	−0.011 (.566)	0.009 (.395)
Mantel cultivar (*p*‐value)^d^	−0.13 (.901)	0.048 (.343)	−0.048 (.531)

a Comparisons made among four species of boxwood. Number represents the test‐statistic from the ANOSIM output followed by the *p*‐value in parentheses.

b Comparisons made among 10 cultivars of *Buxus sempervirens*. Number represents the test‐statistic from the ANOSIM output followed by the *p*‐value in parentheses.

c Test performed comparing distance matrices from microbial taxa with distance matrix from spatial distances among all samples (see Figure 1). Number represents the Mantel coefficient followed by the *p*‐value in parentheses.

d Test performed using samples collected only from *Buxus sempervirens* (*n* = 39).

### 3.3 Impact of boxwood type on microbiome diversity and individual taxon abundance

**Figure 3:**
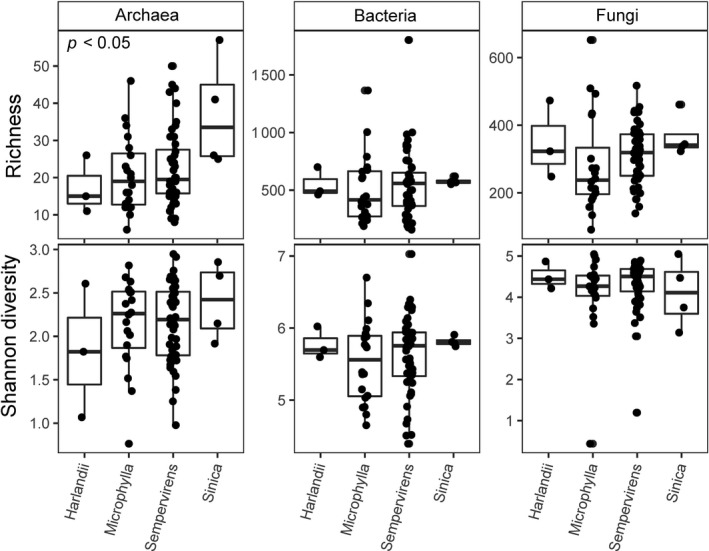
Variation in microbiome diversity among boxwood species. Boxplots show variation in richness and Shannon diversity (*y*‐axis) among soil from four boxwood species (*x*‐axis). Archaeal richness is significantly different among species based on a one‐way ANOVA. Plots without a *p*‐value indicate no significant difference among boxwood species. See main text for further information on statistics and discussion of post hoc comparisons

**Figure 4:**
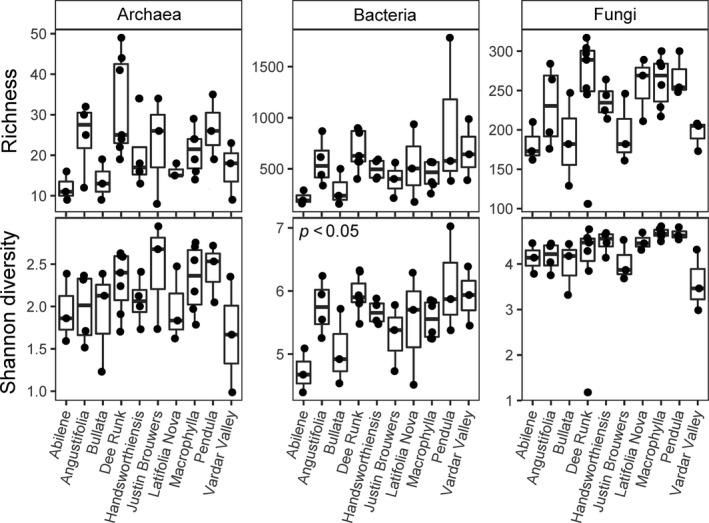
Variation in microbiome diversity among boxwood cultivars. Boxplots show variation in richness and Shannon diversity (*y*‐axis) among soil from ten *Buxus sempervirens* cultivars (*x*‐axis). Bacterial Shannon diversity is significantly different among cultivars based on a one‐way ANOVA. Plots without a *p*‐value indicate no significant difference among cultivars. See main text for further information on statistics and discussion of post hoc comparisons

There were significant differences in microbiome alpha diversity in soil from different boxwood species and *B. sempervirens* cultivars. In particular, archaeal richness was significantly different among boxwood species (*F*_3,71_ = 3.106, *p* < .05; Figure 3). Post hoc comparisons indicated the *B. sinica* samples had significantly higher archaeal richness than *B. microphylla* or *B. sempervirens* samples. Bacterial Shannon diversity was significantly different among the ten cultivars of *B. sempervirens* (*F*_2,29_ = 2.647, *p* < .05; Figure 4). Post hoc comparisons showed the cultivars “Dee Runk” and “Pendula” had significantly higher bacterial Shannon diversity than the cultivar “Abeline”. Overall, these results indicate boxwood type can influence the diversity of different taxonomic components of the soil microbiome.

Boxwood species and cultivar had limited impacts on the abundance of individual microbial taxa. None of the tested bacterial or fungal taxa were significantly influenced by boxwood species or cultivar (data not shown). Similarly, archaeal taxa were not significantly influenced by boxwood cultivar. The single exception to the nonsignificant associations was a single archaeal taxon in the order Nitrosophaerales, which was significantly influenced by boxwood species.

### 3.4 Co‐associations among archaea, bacteria, and fungi

**Figure 5:**
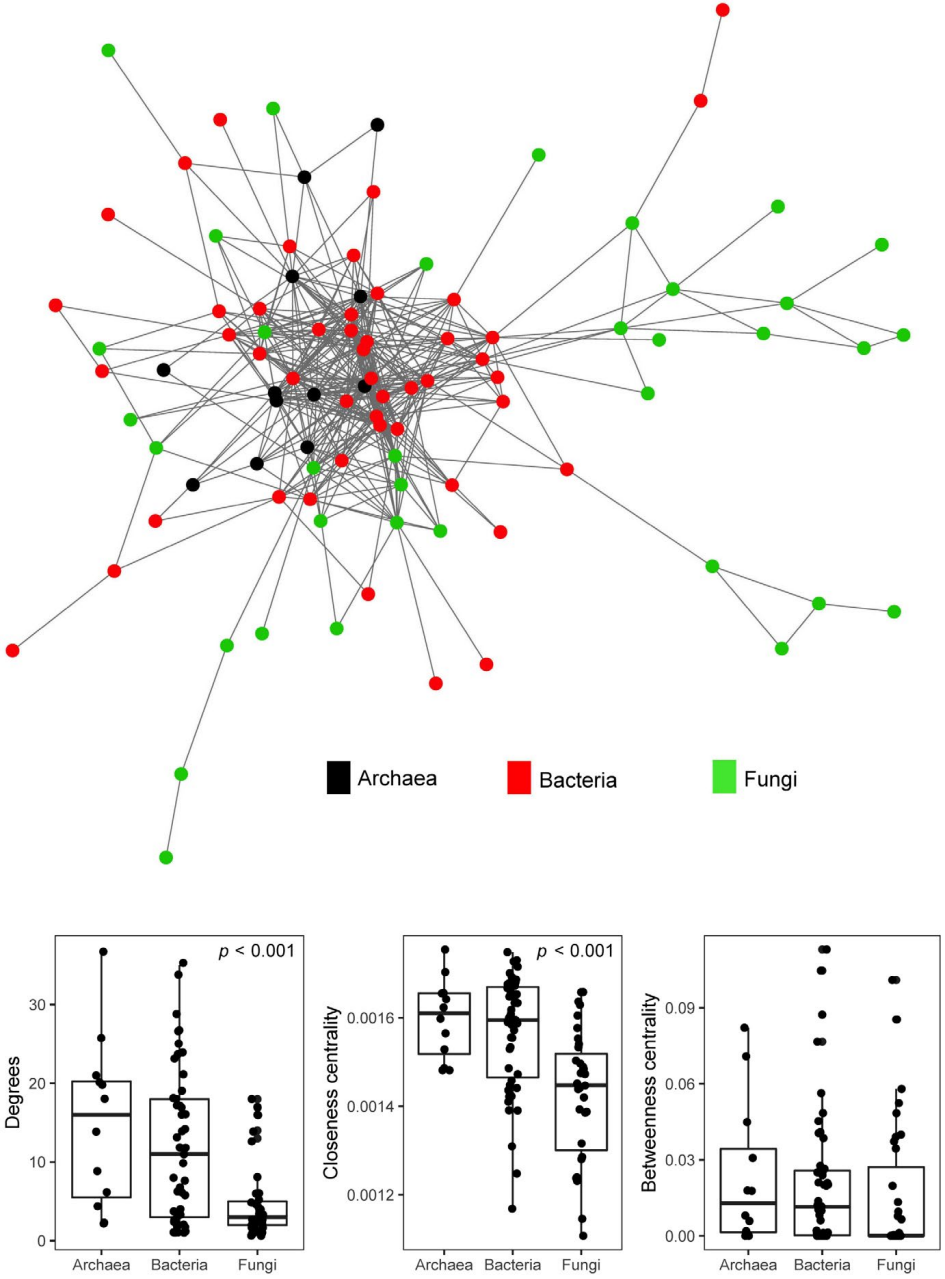
Co‐association network and summary statistics. The network graph represents significant correlations among microbial taxa (nodes), color‐coded by microbial group. Boxplots show three network summary statistics (*y*‐axis) among archaea, bacteria, and fungi (*x*‐axis). Plots without *p*‐values indicate no significant differences among the microbial groups. Network degrees and closeness centrality are significantly greater among archaeal and bacterial taxa than fungi based on TukeyHSD post hoc comparisons

A network graph was generated to quantify co‐associations within and among the three microbial groups as well as to identify keystone taxa. Across the 82 samples, 94 taxa were significantly correlated (Figure 5). As shown in Figure 5, network degrees (*F*_2,91_ = 10.28, *p* < .001) and closeness centrality (*F*_2,91_ = 13.28, *p* < .001) statistics were significantly different among the microbial groups. Post hoc comparisons indicated bacteria and fungi had significantly greater degrees and closeness centrality than archaea (TukeyHSD, *p* < .01). Betweenness centrality was not significantly different among the groups. Within the network, the 12 archaeal taxa were all classified in the order Nitrosophaerales in the phylum Crenarchaeota. The 47 bacterial taxa were classified in five phyla. Rhizobiales was most common bacterial order, represented by 17 taxa. The 35 fungal taxa were classified in the Ascomycota, Basidiomycota, and Zygomycota. The most common fungal order in the network was the Pleosporales, which was represented by 7 taxa.

## 4 DISCUSSION

Despite the promise of using the soil microbiome to improve plant health, little work has focused on soil microbiomes in landscapes predominated by plants cultivated for ornamental purposes. The purpose of this current study was to characterize archaea, bacteria, and fungi as components of the soil microbiome of boxwood germplasm accessions maintained at the U.S. National Arboretum (USNA) National Boxwood Collection. As this observational study was framed within a collection plant accessions with taxonomic designations (Thammina, Olsen, Kramer, & Pooler, 2016), it was possible to evaluate predictors of microbiome composition and test if the association with genetically different boxwood influences individual microbial taxa or the diversity within these microbial groups. In addition, we sought to identify which of these microbial groups shows strongest evidence of containing keystone taxa. Overall, this research provides insight into potential components of the soil microbiome that could be targeted through future management strategies to significantly alter soil microbiome dynamics and provides evidence that the growth of genetically different boxwood in the landscape will impact prokaryotic diversity.

One goal of this work was to test if variation in soil microbiome composition across the USNA National Boxwood Collection was predicted by spatial distance among sample locations or proximity to different types of established boxwood plants. Multiple studies have found evidence that similarity of prokaryotic and eukaryotic microbial communities decreases with increasing spatial distance, suggested to be a result of dispersal limitation (Hanson, Fuhrman, Horner‐Devine, & Martiny, 2012). The growth of different plant species and genotypes is also a known driver of compositional differences among common prokaryotic and eukaryotic microbes in soil (Edwards et al., 2015; LeBlanc, Kinkel, & Kistler, 2017). However, the current study found no evidence that spatial distance or boxwood type were significant predictors of the composition of archaea, bacteria, or fungi in soil. The absence of a relationship between spatial distance and compositional differences of these microbial groups suggests that the predominant taxa within them are not limited to specific areas within the USNA National Boxwood Collection. However, nonlinear relationships between physical distance of the samples and microbiome dissimilarity were not tested. Based on the absence of a strong plant species or cultivar impact, with continued maintenance of the current boxwood accessions and introduction of new germplasm into the National Boxwood Collection, it is unlikely that there will be significant shifts in the overall composition of the soil microbiome. Taken together, these results suggest that efforts to manage keystone taxa and the predominant components of the soil microbiome in this landscape will not be strongly influenced by the physical location or presence of certain types boxwood plants.

Though the overall composition of the soil microbiome was not influenced by the aforementioned factors, there was evidence that presence of different boxwood species and cultivars may influence the diversity of archaea and bacteria in soil. These results raise questions about the nature of these microbiome responses, but also suggest that they could feedback on plant health. The observation that archaeal diversity was significantly different among boxwood species adds to a growing body of evidence that this group of microorganisms may directly interact with plants. Indeed, recent work has found archaea as common endophytes of different olive cultivars (Müller et al., 2015) and part of the heritable portion of the rhizosphere microbiome among maize genotypes (Walters et al., 2018). Despite these examples, aside from their well‐known roles in mediating soil nutrient cycles, it is unknown how variation in archaeal diversity could be leveraged to improve plant health. In contrast, variation in bacterial diversity has been linked to specific functional roles of the soil microbiomes, such as soilborne disease suppression (Gómez Expósito, Bruijn, Postma, & Raajimakers, 2017; Hu et al., 2016; Mehrabi et al., 2016). Consequently, the observed variation in bacterial diversity suggests microbiome‐mediated pathogen suppression may differ in soil under the influence of different types of boxwood at the USNA. Future work will need to evaluate if these boxwood type effects are reproducible across soil types or variable environments when the plants are under pressure from diseases like boxwood blight or infected with insect pests.

Another goal of this work was to identify which of the three targeted taxonomic groups showed greatest evidence of being keystone taxa in the soil microbiome at the USNA. Through analysis of co‐association networks, we found that archaea and bacteria showed greater statistical evidence of being keystone taxa than fungi. This conclusion was supported by the observation that prokaryotes have a greater number of degrees and higher closeness centrality metrics than fungi, both of which are indicators of keystone taxa (Berry & Widder, 2014) or predictors of “hub taxa” (Agler et al., 2016). Among prokaryotic taxa, the archaeal order Nitrosophaerales and bacterial order Rhizobiales were the most common in the co‐association network, showing similarities with other studies (Barberán, Bates, Casamayor, & Fierer, 2012; Bonito et al., 2019). Bacteria in the Rhizobiales have primarily been studied for their ability to fix atmospheric nitrogen as symbionts in roots of leguminous plants. However, there is growing evidence that nonsymbiotic Rhizobiales are abundant in rhizosphere and can have additional functional impacts on plant physiology (Garrido‐Oter et al., 2018). Archaea in the Nitrosophaerales are common in soil (Bates et al., 2011). Based on biochemical and genomic data, these microorganisms are thought to play a role in the oxidation of ammonia (Stahl & de la Torre, 2012), a key step in the soil nitrogen cycle. Considering this physiological trait, the high number of correlations these taxa showed with the rest of the microbiome, including the Rhizobiales, could be due to their ability to alter the type of nitrogen in soil. The practical outcomes of these results are that managing these ammonia‐oxidizing archaea through cultural practices like fertilization (Xue et al., 2016) could indirectly influence the functional activity of the broader soil microbiome at the USNA.

Archaeal, bacterial, and fungal components of the soil microbiome are not often characterized in the same study. However, the growing trend derived from studies that have looked at these three microbial groups is that ecological predictors of their diversity and composition are not equivalent. For example, differences have been found among these groups when characterizing their seasonal variation (Pereira e Silva, Dias, Elsas, & Salles, 2012), correlations with soil physiochemical characteristics (Peay et al., 2017), and continental‐scale biogeographic patterns (Ma, Dai, et al., 2016). At the same time, the co‐associations among archaea, bacteria, and fungi demonstrated in this and other (Ma, Wang, et al., 2016) research highlight the potential for these three groups to influence the activity of each other in soil across the landscape. Consequently, understanding and managing soil or host‐microbiomes to improve plant health through specific mechanisms like disease suppression should benefit from a broader sampling of the different microbial groups found in soil.

This work provides a first step toward addressing the significant lack of research on the soil microbiome of landscapes harboring ornamental plants. Though there are limitations specific to this work (e.g., the data are observational and it was not possible to collect soil physiochemical data), the primary constraints are also reflected in the majority of microbiome studies. Namely, the complex nature of microbiomes limits our ability to identify which microbiome components are actually interacting and how these interactions influence host health. As advocated elsewhere (Busby et al., 2017; Vorholt, Vogel, Carlström, & Müller, 2017), future work in ornamental systems should pair meta‐barcoding surveys with experimentation using simplified microbial communities to understand the causal relationships among inferred microbial interactions and how they feedback on host health. Once these simplified communities are understood, gradually reintroducing the microbial diversity observed in nature and use of controlled field experiments will help develop a better framework for managing microbiomes for promoting ornamental plant health.

## CONFLICT OF INTEREST

None declared.

## AUTHOR CONTRIBUTIONS

JAC and NL conceived the study. NL generated, analyzed, and interpreted the data. JAC and NL drafted and revised the final manuscript.

## Data Availability

Sequence data are deposited and available at NCBI's Sequence Read Archive as BioProject accession PRJNA503120.

## References

[ece35614-bib-0001] Agler, M. T. , Ruhe, J. , Kroll, S. , Morhenn, C. , Kim, S. T. , Wiegel, D. , & Kemen, E. M. (2016). Microbial hub taxa link host and abiotic factors to plant microbiome variation. PLoS Biology, 14, e1002352 10.1371/journal.pbio.1002352 26788878 PMC4720289

[ece35614-bib-0002] Bakker, M. G. , Manter, D. K. , Sheflin, A. M. , Weir, T. L. , & Vivanco, J. G. (2012). Harnessing the rhizosphere microbiome through plant breeding and agricultural management. Plant and Soil, 360, 1–13. 10.1007/s11104-012-1361-x

[ece35614-bib-0003] Barberán, A. , Bates, S. T. , Casamayor, E. O. , & Fierer, N. (2012). Using network analysis to explore co‐occurrence patterns in soil microbial communities. The ISME Journal, 6, 343–351. 10.1038/ismej.2011.119 21900968 PMC3260507

[ece35614-bib-0004] Batdorf, L. (2004). Boxwood and illustrated encyclopedia. Boyce, VA: American Boxwood Society.

[ece35614-bib-0005] Bates, S. T. , Berg‐Lyons, D. , Caporaso, J. G. , Walters, W. A. , Knight, R. , & Fierer, N. (2011). Examining the global distribution of dominant archaeal populations in soil. The ISME Journal, 5, 908–917. 10.1038/ismej.2010.171 21085198 PMC3105767

[ece35614-bib-0006] Beirn, L. A. , Hempfling, J. W. , Schmid, C. J. , Murphey, J. A. , Clarcke, B. B. , & Crouch, J. A. (2017). Differences among soil‐inhabiting microbial communities in *Poa annua* turf throughout the growing season. Crop Science, 57, S262–S273.

[ece35614-bib-0007] Berry, D. , & Widder, S. (2014). Deciphering microbial interactions and detecting keystone species with co‐occurrence networks. Frontiers in Microbiology, 5, 219 10.3389/fmicb.2014.00219 24904535 PMC4033041

[ece35614-bib-0008] Bonito, G. , Benucci, G. M. N. , Hameed, K. , Weighill, D. , Jones, P. , Chen, K. H. , … Vilgalys, R. (2019). Fungal‐bacterial networks in the *Populus* rhizobiome are impacted by soil properties and host genotype. Frontiers in Microbiology, 10, 481 10.3389/fmicb.2019.00481 30984119 PMC6450171

[ece35614-bib-0009] Busby, P. E. , Soman, C. , Wagner, M. R. , Friesen, M. L. , Kremer, J. , Bennet, A. , … Dangl, J. L. (2017). Research priorities for harnessing plant microbiomes in sustainable agriculture. PLoS Biology, 15, e2001793 10.1371/journal.pbio.2001793 28350798 PMC5370116

[ece35614-bib-0010] Callahan, B. J. , McMurdie, P. J. , & Holmes, S. P. (2017). Exact sequence variants should replace taxonomic units in marker‐gene data analysis. The ISME Journal, 11, 2639–2643. 28731476 10.1038/ismej.2017.119 PMC5702726

[ece35614-bib-0011] Callahan, B. J. , McMurdie, P. J. , Rosen, M. J. , Han, A. W. , Johnson, A. J. A. , & Holmes, S. P. (2016). DADA2: High‐resolution sample inference from Illumina amplicon data. Nature Methods, 13, 581–583. 10.1038/nmeth.3869 27214047 PMC4927377

[ece35614-bib-0012] Crouch, J. A. , Carter, Z. , Ismaiel, A. , & Roberts, J. A. (2017). The U.S. National Mall microbiome: A census of rhizosphere bacteria inhabiting landscape turf. Crop Science, 57, S341–S348.

[ece35614-bib-0013] d'Eustachio, G. , & Raupp, M. (2001). Resistance of boxwood varieties to the boxwood leafminer, *Monarthropalpus flavus* (Schrank). Journal of Environmental Horticulture, 19, 153–157.

[ece35614-bib-0014] Domenico, F. D. , Lucchese, F. , & Magri, D. (2012). Buxus in Europe: Late quaternary dynamics and modern vulnerability. Perspectives in Plant Ecology, Evolution and Systematics, 14, 354–362. 10.1016/j.ppees.2012.07.001

[ece35614-bib-0015] Edwards, J. , Johnson, C. , Santos‐Medellín, C. , Lurie, E. , Podishetty, N. K. , Bhatnagar, S. , … Sundaresan, V. (2015). Structure, variation and assembly of the root associated microbiomes of rice. Proceedings of the National Academy of Sciences of the United States of America, 112, E911–E920. 10.1073/pnas.1414592112 25605935 PMC4345613

[ece35614-bib-0016] Fierer, N. (2017). Embracing the unknown: Disentangling the complexities of the soil microbiome. Nature Reviews Microbiology, 15, 579–590. 10.1038/nrmicro.2017.87 28824177

[ece35614-bib-0017] Friedman, J. , & Alm, E. J. (2012). Inferring correlation networks from genomic survey data. PLoS Computational Biology, 8, e1002687 10.1371/journal.pcbi.1002687 23028285 PMC3447976

[ece35614-bib-0018] Gabor, C. , & Nepusz, T. (2006). The igraph software package for complex network research. InterJournal Complex Systems:1695. Retrieved from http://www.interjournal.org/manuscript_abstract.php?361100992

[ece35614-bib-0019] Garrido‐Oter, R. , Nakano, R. T. , Dombrowski, N. , Ma, K.‐W. , Team, T. A. B. , McHardy, A. C. , & Schulze‐Lefert, P. (2018). Modular traits of the Rhizobiales root microbiota and their evolutionary relationship with symbiotic rhizobia. Cell Host & Microbe, 24, 155–167. 10.1016/j.chom.2018.06.006 30001518 PMC6053594

[ece35614-bib-0020] Gómez Expósito, R. , de Bruijn, I. , Postma, J. , & Raajimakers, J. M. (2017). Current insights into the role of rhizosphere bacteria in disease suppressive soils. Frontiers in Microbiology, 8, 3389 10.3389/fmicb.2017.02529 PMC5741648 29326674

[ece35614-bib-0021] Hanson, C. A. , Fuhrman, J. A. , Horner‐Devine, M. C. , & Martiny, J. B. H. (2012). Beyond biogeographic patterns. Nature Reviews Microbiology, 10, 497–506. 22580365 10.1038/nrmicro2795

[ece35614-bib-0022] Hu, J. , Wei, Z. , Friman, V.‐P. , Gu, S.‐H. , Wang, X.‐F. , Eisenhauer, N. , … Jousset, A. (2016). Probiotic diversity enhances rhizosphere microbiome function and plant disease suppression. Mbio, 7, e01790‐16 10.1128/mBio.01790-16 27965449 PMC5156302

[ece35614-bib-0024] Kenis, M. , Nacambo, S. , Leuthardt, F. L. G. , Di Domenico, F. , & Haye, T. (2013). The box tree moth, *Cydalima perspectalis*, in Europe: Horticultural pest or environmental disaster? Aliens, 33, 38–41.

[ece35614-bib-0025] Knight, R. , Vrbanac, A. , Taylor, B. C. , Aksenov, A. , Callewaert, C. , Debelius, J. , … Dorrestein, P. C. (2018). Best practices for analyzing microbiomes. Nature Reviews Microbiology, 16, 410–422. 29795328 10.1038/s41579-018-0029-9

[ece35614-bib-0027] Lamondia, J. A. , & Shishkoff, N. (2017). Susceptibility of boxwood accessions from the National Boxwood Collection to boxwood blight and potential for differences between *Calonectria pseudonaviculata* and *C. henricotiae* . HortScience, 52, 873–879. 10.21273/HORTSCI11756-17

[ece35614-bib-0028] LeBlanc, N. , Kinkel, L. , & Kistler, H. C. (2017). Plant diversity and identity influence *Fusarium* communities in soil. Mycologia, 109, 128–139. 28402790 10.1080/00275514.2017.1281697

[ece35614-bib-0029] LeBlanc, N. , Salgado‐Salazar, C. , & Crouch, J. A. (2018). Boxwood blight: An ongoing threat to ornamental and native boxwood. Applied Microbiology and Biotechnology, 102, 4371–4380. 10.1007/s00253-018-8936-2 29610965 PMC5932091

[ece35614-bib-0030] Love, M. I. , Huber, W. , & Anders, S. (2014). Moderated estimation of fold change and dispersion for RNA‐seq data with DESeq2. Genome Biology, 15, 550 10.1186/s13059-014-0550-8 25516281 PMC4302049

[ece35614-bib-0031] Ma, B. , Dai, Z. , Wang, H. , Dsouza, M. , Liu, X. , He, Y. , … Xu, J. (2016). Distinct biogeographic patterns for archaea, bacteria, and fungi along the vegetation gradient at the continental scale in eastern China. mSystems, 2, e00174‐16 https://doi.org/10.1128.mSystems.00174-16 10.1128/mSystems.00174-16 PMC5296412 28191504

[ece35614-bib-0032] Ma, B. , Wang, H. , Dsouza, M. , Lou, J. , He, Y. , Brookes, P. C. , … Gilbert, J. A. (2016). Geographic patterns of co‐occurrence network topological features for soil microbiota at continental scale in eastern China. The ISME Journal, 10, 1891–1901. 26771927 10.1038/ismej.2015.261 PMC5029158

[ece35614-bib-0033] Mehrabi, Z. , McMillan, V. E. , Clark, I. M. , Canning, G. , Hammond‐Kosack, K. E. , Preston, G. , … Mauchline, T. H. (2016). *Pseudomonas* spp. diversity is negatively associated with suppression of the wheat take‐all pathogen. Scientific Reports, 6, 29905. 27549739 10.1038/srep29905 PMC4993996

[ece35614-bib-0034] Müller, H. , Berg, C. , Landa, B. B. , Auerbach, A. , Moissl‐Eichinger, C. , & Berg, G. (2015). Plant genotype‐specific archaeal and bacterial endophytes colonize Mediterranean olive trees. Frontiers in Microbiology, 6, 138. 25784898 10.3389/fmicb.2015.00138 PMC4347506

[ece35614-bib-0035] Niemiera, A. X. (2012). Selecting landscape plants: Boxwoods. Retrieved from https://pubs.ext.vt.edu/content/dam/pubs_ext_vt_edu/426/426-603/HORT-290.pdf

[ece35614-bib-0036] Oksanen, J. , Blanchet, F. G. , Friendly, M. , Kindt, R. , Legendre, P. , McGlinn, D. , … Szoecs, E. (2018). Vegan: Community ecology package. R package version 2.5‐2. Retrieved from https://CRAN.R-project.org/package=vegan

[ece35614-bib-0037] Palmer, C. L. , & Shishkoff, N. (2014). Boxwood blight: A new scourge, a new paradigm for collaborative research. Outlooks on Pest Management, 25, 230–236.

[ece35614-bib-0038] Peay, K. G. , von Sperber, C. , Toju, H. , Francis, C. A. , Chadwick, O. A. , & Vitousek, P. M. (2017). Convergence and contrast in the community structure of Bacteria, Fungi and Archaea along a tropical elevation‐climate gradient. FEMS Microbiology Ecology, 93, 1–12. 10.1093/femsec/fix045 28402397

[ece35614-bib-0040] Pereira e Silva, M. C. , Dias, A. C. , van Elsas, J. D. , & Salles, J. F. (2012). Spatial and temporal variation of archaeal, bacterial and fungal communities in agricultural soils. PLoS ONE, 7, e51554. 23284712 10.1371/journal.pone.0051554 PMC3527478

[ece35614-bib-0041] Pooler, M. (2017). Good genes – using germplasm and breeding to create new plants at the U.S. National Arboretum. ISHS Acta Horticulturae 1185: II International Symposium on Germplasm of Ornamentals, 1–6. 10.17660/ActaHortic.2017.1185.1

[ece35614-bib-0042] R Development Core Team (2015). R: A language and environment for statistical computing. Vienna, Austria: R Foundation for Statistical Computing.

[ece35614-bib-0043] Schloerke, B. , Crowley, J. , Cook, D. , Briatte, F. , Marbach, M. , Thoen, E. , … Larmarange, J. (2018). GGaley: Extension to ‘ggplot2’. R package version 1.4.0. Retrieved from https://CRAN.R-project.org/package=GGally

[ece35614-bib-0044] Schloss, P. D. , Westcott, S. L. , Ryabin, T. , Hall, J. R. , Hartmann, M. , Hollister, E. B. , … Weber, C. F. (2009). Introducing mothur: Open‐source, platform‐independent, community‐supported software for describing and comparing microbial communities. Applied and Environment Microbiology, 75, 7537–7541. 10.1128/AEM.01541-09 PMC2786419 19801464

[ece35614-bib-0045] Shishkoff, N. , Daughtrey, M. , Aker, S. , & Olsen, R. T. (2015). Evaluating boxwood susceptibility to *Calonectria pseudonaviculata* using cuttings from the National Boxwood Collection. Plant Health Progress, 16, 11–15.

[ece35614-bib-0046] Stahl, D. A. , & de la Torre, J. R. (2012). Physiology and diversity of ammonia‐oxidizing archaea. Annual Review of Microbiology, 66, 83–101. 10.1146/annurev-micro-092611-150128 22994489

[ece35614-bib-0047] Thammina, C. S. , Olsen, R. T. , Kramer, M. , & Pooler, M. R. (2016). Genetic relationships of boxwood (*Buxus* L.) based on genic simple sequence repeat markers. Genetic Resources and Crop Evolution, 64, 1281–1293.

[ece35614-bib-0048] Vorholt, J. A. , Vogel, C. , Carlström, C. I. , & Müller, D. B. (2017). Establishing causality: Opportunities of synthetic communities for plant microbiome research. Cell Host & Microbe, 22, 142–155. 10.1016/j.chom.2017.07.004 28799900

[ece35614-bib-0049] Walters, W. A. , Zhao, J. , Youngblut, N. , Wallace, J. G. , Sutter, J. , Zhang, W. , … Ley, R. E. (2018). Large‐scale replicated field study of maize rhizosphere identifies heritable microbes. Proceedings of the National Academy of Sciences of the United States of America, 28, 7368–7373. 10.1073/pnas.1800918115 PMC6048482 29941552

[ece35614-bib-0051] Wang, Q. , Garrity, G. M. , Tiedje, J. M. , & Cole, J. R. (2007). Naïve Bayesian classifier for rapid assignment of rRNA sequences into the new bacterial taxonomy. Applied and Environment Microbiology, 73, 5261–5267. 10.1128/AEM.00062-07 PMC1950982 17586664

[ece35614-bib-0053] Xue, C. , Zhang, X. , Zhu, C. , Zhao, J. , Zhu, P. , Peng, C. , … Shen, Q. (2016). Quantitative and compositional responses of ammonia‐oxidizing archaea and bacteria to long‐term field fertilization. Scientific Reports, 6, 28981 10.1038/srep28981 27356769 PMC4928058

