# Supplementary material for: Prokaryotic taxa play keystone roles in the soil microbiome associated with woody perennial plants in the genus Buxus
Source: Ecol Evol. 2019 Aug 26;9(19):11102–11. doi: 10.1002/ece3.5614 (PMC6802073; doi:10.1002/ece3.5614)
Supplement: Supplementary file 1 [file ECE3-9-11102-s001.pdf]

Supplementary Table 1 Individual boxwood accessions targeted for soil sampling at the U.S. National Arboretum (USNA) National Boxwood Collection.

USNA_Accession[a]	Species[b]	Cultivar[c]	NCBI_BioSample_Accession
16331CL	sempervirens	Latifolia Nova	SAMN10352813
16477K	sempervirens	Angustifolia	SAMN10352814
17084H	sempervirens	Bullata	SAMN10352815
17089J	sempervirens	Latifolia Nova	SAMN10352816
17089K	sempervirens	Latifolia Nova	SAMN10352817
17090H	sempervirens	Angustifolia	SAMN10352818
17093CH	sempervirens	Handsworthiensis	SAMN10352819
17525CH	-	-	SAMN10352820
17525CJ	-	-	SAMN10352821
17525CL	-	-	SAMN10352822
17525K	-	-	SAMN10352823
26393H	sempervirens	Joe Gable	SAMN10352824
31791H	sempervirens	-	SAMN10352825
31791J	sempervirens	-	SAMN10352826
31792H	sempervirens	-	SAMN10352827
31796H	sempervirens	-	SAMN10352828
33811H	-	seedling selection	SAMN10352829
33811J	-	seedling selection	SAMN10352830
33812H	microphylla	seedling selection	SAMN10352831
33901H	microphylla	Green Pillow	SAMN10352832
33901J	microphylla	Green Pillow	SAMN10352833
33901K	microphylla	Green Pillow	SAMN10352834
33901L	microphylla	Green Pillow	SAMN10352835
33902J	microphylla	-	SAMN10352836
33904H	sempervirens	Justin Brouwers	SAMN10352837
33904J	sempervirens	Justin Brouwers	SAMN10352838
33904V	sempervirens	Justin Brouwers	SAMN10352839
34083H	-	hybrid	SAMN10352840
34593H	sempervirens	Abilene	SAMN10352841
34593J	sempervirens	Abilene	SAMN10352842
34593K	sempervirens	Abilene	SAMN10352843
35490H	sempervirens	Bullata	SAMN10352844
35490J	sempervirens	Bullata	SAMN10352845
36672H	harlandii	-	SAMN10352846
41758H	sinica	var. insularis Winterg	SAMN10352847
4204H	microphylla	-	SAMN10352848
4204K	microphylla	-	SAMN10352849
4207H	sempervirens	Macrophylla	SAMN10352850
4207K	sempervirens	Macrophylla	SAMN10352851
4207L	sempervirens	Macrophylla	SAMN10352852
4207P	sempervirens	Macrophylla	SAMN10352853
4207R	sempervirens	Macrophylla	SAMN10352854
4207T	sempervirens	Macrophylla	SAMN10352855
4224H	sempervirens	Angustifolia	SAMN10352856
4224J	sempervirens	Angustifolia	SAMN10352857
4227R	microphylla	-	SAMN10352858
4233H	sempervirens	Handsworthiensis	SAMN10352859
4233J	sempervirens	Handsworthiensis	SAMN10352860
4233K	sempervirens	Handsworthiensis	SAMN10352861
4899CH	microphylla	Compacta KINGSVILL	SAMN10352862
4899CJ	microphylla	Compacta KINGSVILL	SAMN10352863
4899J	microphylla	Compacta KINGSVILL	SAMN10352864
51896H	sempervirens	-	SAMN10352865
51896T	sempervirens	-	SAMN10352866
51898H	sinica	var. insularis Pincush	SAMN10352867
51898J	sinica	var. insularis Pincush	SAMN10352868
51900H	sinica	var. insularis Winter	SAMN10352869
59820J3	sempervirens	Pendula	SAMN10352870
59820T	sempervirens	Pendula	SAMN10352871
59820X	sempervirens	Pendula	SAMN10352872
6395H	sempervirens	Vardar Valley	SAMN10352873
6395K	sempervirens	Vardar Valley	SAMN10352874
68631H	sempervirens	Dee Runk	SAMN10352875
68631J	sempervirens	Dee Runk	SAMN10352876
68631P	sempervirens	Dee Runk	SAMN10352877
7025H	microphylla	National	SAMN10352878

7025J	microphylla	National	SAMN10352879
7025R	microphylla	National	SAMN10352880
7025U	microphylla	National	SAMN10352881
7025V	microphylla	National	SAMN10352882
7026J3	microphylla	Morris Midget	SAMN10352883
7026J	microphylla	Morris Midget	SAMN10352884
7026V	microphylla	Morris Midget	SAMN10352885
7030K	sempervirens	Salicifolia Elata	SAMN10352886
7030P	sempervirens	Salicifolia Elata	SAMN10352887
7032H	harlandii	-	SAMN10352888
7032P	harlandii	-	SAMN10352889
7034CR	sempervirens	Vardar Valley	SAMN10352890
80313J2	sempervirens	Dee Runk	SAMN10352891
80313J	sempervirens	Dee Runk	SAMN10352892
80313T	sempervirens	Dee Runk	SAMN10352893
80313V	sempervirens	Dee Runk	SAMN10352894

[a]Unique barcode assigned to individual boxwood plants at the USNA National Boxwood Collection.

[b]Species designation of boxwood accessions. A dash indicates an absence of classification by the USNA.

[c]Cultivar designation of boxwood accessions. A dash indicates an absence of classification by the USNA.
