# Supplementary material for: Prokaryotic taxa play keystone roles in the soil microbiome associated with woody perennial plants in the genus Buxus
Source: Ecol Evol. 2019 Aug 26;9(19):11102–11. doi: 10.1002/ece3.5614 (PMC6802073; doi:10.1002/ece3.5614)
Supplement: Supplementary file 2 [file ECE3-9-11102-s002.pdf]

Supplementary Table 2 Primer sequences used to construct amplicon libraries targeting archaea, bacteria, and fungi.

Primer	Sequence(5'-3')[a]	Target	Group	Reference
Ar915aF	AGGAATTGGCGGGGGAGCAC	16S rRNA	Archaea	Kittelmann et al. 2013
Ar1386R	GCGGTGTGTGCAAGGAGC	16S rRNA	Archaea	Kittelmann et al. 2013
Ba9F	GAGTTTGATCMTGGCTCAG	16S rRNA	Bacteria	Kittelmann et al. 2013
Ba515Rmod1	CCGCGGCKGCTGGCAC	16S rRNA	Bacteria	Kittelmann et al. 2013
ITS3_KYO2	GATGAAGAACGYAGYRAA	ITS2	Fungi	Toju et al. 2012
ITS4	TCCTCCGCTTATTGATATGC	ITS2	Fungi	Toju et al. 2012

[a]Forward and reverse primers were appended at the 5' ends with Illumina specific sequences as described in Beirn et al. 2017.

References

Beirn, L. A., Hempfling, J. W., Schmid, C. J., Murphey, J. A., Clarcke, B. B., Crouch, J. A. 2017. Crop Sci. 57:S262-S273.
Kittelmann, S., Seedorf, H., Walters, W. A., Clemente, J. C., Knight, R., Gordon, J. I., Janssen, P. H. 2013. PLoS One 8:e47879.
Toju, H., Tanabe, A. S., Yamamoto, S., Sato, H. 2012. PLoS One 7: e40863.
